# Nanoscale remodeling of mitochondrial architecture during mtRNA surveillance failure

**DOI:** 10.64898/2026.10.20.759735

**Published:** 2026-10-21

**Authors:** Eve V. Kakudji, Kevin Rose, Jaewon Lee, Eric Herrmann, Priya Gatti, James H. Hurley, Samantha C. Lewis

## Abstract

Mitochondria within a cell exhibit heterogeneity in their composition and metabolic functions, and how this specialization shapes the responses of individual organelles to stress is unclear. We applied fluorescence microscopy and *in situ* cryo-electron tomography to examine the composition of mitochondrial stress bodies and their relationship to inner mitochondrial membrane architecture in the context of a mitochondrial genome expression defect. We discovered that defective mitochondrial RNA processing prompted a loss of inner membrane domain identity, leading to the mislocalization of ATP synthase from cristae edges to the boundary membrane, perturbed cristae morphometrics, and complete cristae loss in a subset of organelles. Mitochondrial stress bodies were enriched with nucleic acid and protein components linked to oxidative pathways, while separate and distinct mitochondrial domains became enriched with oligomerized P5CS, a marker of reductive proline synthesis. We found that consolidation of mitochondrial RNA into stress bodies, as well as ATP synthase remodeling, were regulated independently from P5CS concentration into a subset of the mitochondria. We propose that stress compartmentalization enables independent tuning of the quality control of oxidative pathway components via spatial sequestration from the rest of the mitochondrial network.

